# Open and closed evolutionary paths for drastic morphological changes, involving serial gene duplication, sub-functionalization, and selection

**DOI:** 10.1038/srep26838

**Published:** 2016-05-25

**Authors:** Gembu Abe, Shu-Hua Lee, Ing-Jia Li, Chun-Ju Chang, Koji Tamura, Kinya G. Ota

**Affiliations:** 1 Laboratory of Aquatic Zoology, Marine Research Station, Institute of Cellular and Organismic Biology, Academia Sinica, Yilan, 26242, Taiwan; 2 Laboratory of Organ Morphogenesis, Department of Developmental Biology and Neurosciences, Graduate School of Life Sciences, Tohoku University, Aobayama Aoba-ku, Sendai 980-8578, Japan

## Abstract

Twin-tail goldfish strains are examples of drastic morphological alterations that emerged through domestication. Although this mutation is known to be caused by deficiency of one of two duplicated *chordin* genes, it is unknown why equivalent mutations have not been observed in other domesticated fish species. Here, we compared the *chordin* gene morphant phenotypes of single-tail goldfish and common carp (close relatives, both of which underwent *chordin* gene duplication and domestication). Morpholino-induced knockdown depleted *chordin* gene expression in both species; however, while knockdown reproduced twin-tail morphology in single-tail goldfish, it had no effect on common carp morphology. This difference can be explained by the observation that expression patterns of the duplicated *chordin* genes overlap completely in common carp, but are sub-functionalized in goldfish. Our finding implies that goldfish drastic morphological changes might be enhanced by the subsequent occurrence of three different types of evolutionary event (duplication, sub-functionalization, and selection) in a certain order.

Domesticated animals have provided some important insights into how morphological features can be altered during the course of evolution^1^,^2^,^3^,^4^. In particular, highly diverged ornamental and pet animals have provided evidence that artificial selection can drastically change morphological features^1^,^3^,^4^; moreover, recent genomic studies of domesticated animals have identified certain genes responsible for the morphological changes that emerged during artificial selection^3^,^4^. The twin-tail phenotype of goldfish, which was established by artificial selection of wildtype (single-tail) *Carassius auratus* species, is one such representative example^5^,^6^,^7^,^8^,^9^ (Fig. 1a–c). Our previous molecular developmental genetic study revealed that a stop codon mutation in one of two *chordin* paralogous genes (*chdA* and *-B*, which arose from a recent duplication event prior to domestication) is responsible for twin-tail morphology; the stop codon allele located in *chdA* is designated as *chdA*^*E127X*^ (Fig. 1a,c and Supplementary Fig. S1)^9^. On the basis that the *chordin* gene plays a crucial role in dorsal-ventral patterning^10^,^11^,^12^,^13^,^14^,^15^,^16^, it is presumed that in the absence of the *chordin* gene duplication, it may be difficult (if not impossible) for the equivalent morphological mutation to be genetically fixed, even in domesticated populations (Fig. 1a); in fact, stably fixed twin-tail strains/species have not been reported for any other vertebrate so far^8^,^9^.

However, we are left with the following question: why has twin-tail morphology not been found in other fish species which underwent both the same *chordin* gene duplication event and domestication for ornamental purposes? For example, common carp (*Cyprinus carpio*, the closest relative of goldfish) has *chdA* and *-B* orthologues which were acquired by allotetraploidization (species hybrid polyploidization) (Fig. 1a,d,e and Supplementary Fig. S2)^17^. Moreover, common carp is one of the most popular ornamental fish species among breeders worldwide (Fig. 1d)^7^,^18^. Given the anticipated commercial value of “twin-tail common carp” as an ornamental fish, it can be assumed that breeders would be keen to genetically fix this morphological mutation in breeding populations. Although there is more recent reliable documentation on ornamental common carp breeding history than there is for ornamental goldfish (Fig. 1a)^7^,^18^,^19^, there are no records of the appearance of twin-tail morphology in the common carp lineage; we should note that other ornamentation-related phenotypes (for example, favorable fur color and texture, body size, and tameness) have been independently reproduced in different vertebrate lineages during relatively short periods of domestication^2^,^3^,^4^. The absence of twin-tail carp despite clear incentives for breeders to select for such organisms strongly suggests that this morphological mutation does not occur readily in this species, prompting us to investigate why this mutation arose in goldfish, but not in the closely related common carp (Fig. 1a).

Previous examinations of *dino/chordin* zebrafish^10^ and backcross analysis of twin-tail goldfish^9^ revealed that the phenotypes of these *chordin* gene mutants are polymorphic. In fact, a few F2 segregants of *chdA*^*E127X/E127X*^ derived from our backcross analysis did not show any mutated phenotypes at the late embryonic stage^9^, due to incomplete penetrance presumably stemming from the robustness of dorsal-ventral patterning^9^,^15^,^16^. Moreover, we observed that the mutated embryonic caudal fin fold of *chdA*^*E127X/E127X*^ goldfish individuals can revert to a more wild-type morphology, and the restored fin fold ultimately forms a wild-type caudal fin at the post-embryonic stage (Supplementary Fig. S3)^9^. Based on such evidence, we hypothesize that the presence or absence of twin-tail morphology is related to how embryos respond to the *chordin* gene-deficient condition. To explore this hypothesis, we here analyzed phenotypes of single and double *chordin* gene morphants of wildtype and twin-tail goldfish, and of common carp (Fig. 1 and Supplementary Fig. S1).

## Results

### Phenotype analysis of *chordin* gene-deficient condition in goldfish

**Figure 1:**
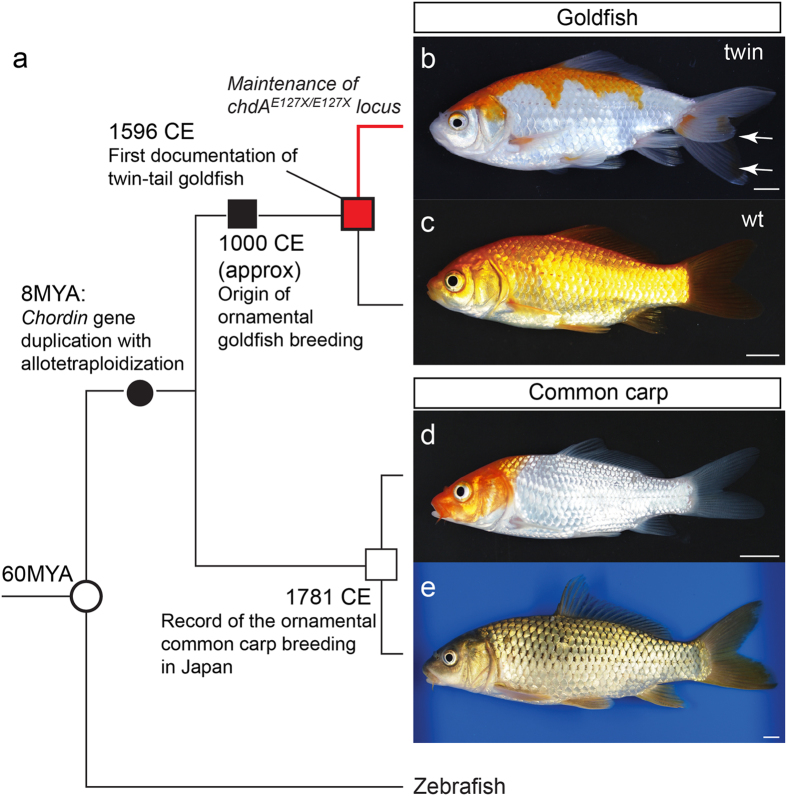
Relationship between goldfish and common carp. (**a**) Phylogenetic tree and historical/evolutionary events. The branch lengths are arbitrary and do not reflect evolutionary time. Red, black, and white squares indicate the first records of twin-tail goldfish, the approximate origin of goldfish domestication for ornamental purposes, and the first records of ornamental common carp breeding in Japan, respectively. Black and white circles indicate the allotetraploidization event and divergence of zebrafish and other teleost lineages (goldfish and common carp), respectively. The evolutionary relationship and events are based on refs 17, 18, 19,36,40,41. The red line indicates the twin-tail goldfish lineage. Photographs on right: (**b**) Twin-tail goldfish (*Mitsuo-wakin* strain). White arrows indicate the bifurcated caudal fin. (**c**) Wildtype (single-tail) goldfish. (**d**) Ornamental common carp. (**e**) Wildtype common carp. Scale bar = 1 cm.

**Figure 2:**
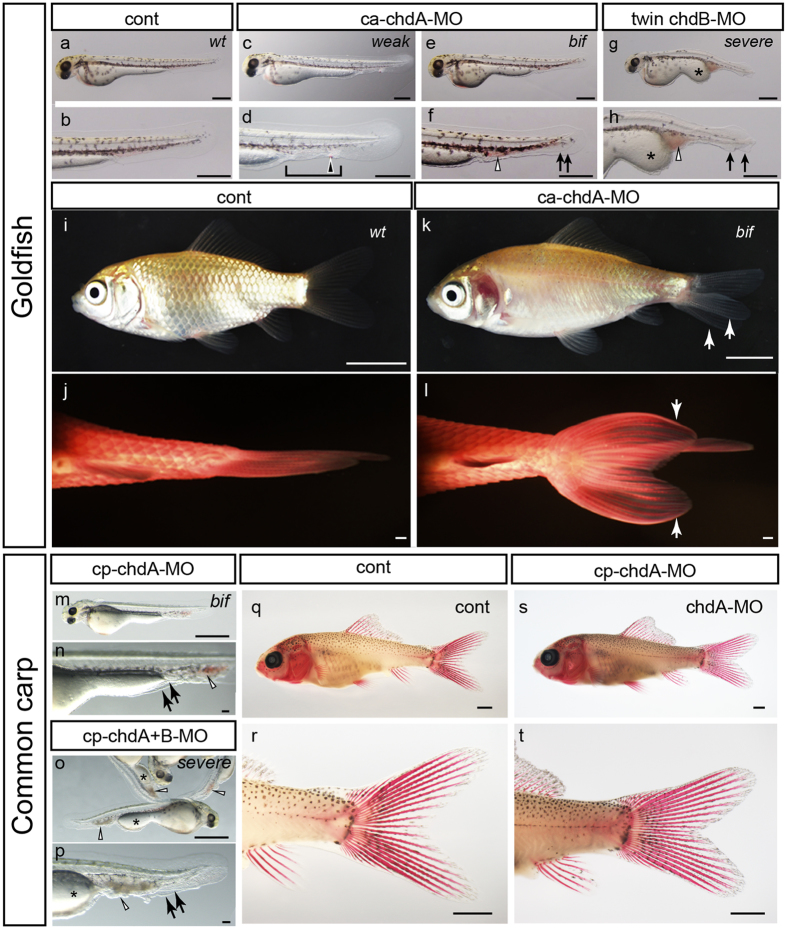
Comparison of phenotypes between goldfish and common carp. (**a**–**h**) Late-stage goldfish embryos (**a**,**b**: control; **c**–**h**: *chordin* gene deficient embryos; *wt* (wildtype), *weak* (weakly ventralized), *bif* (bifurcated fin fold), and *severe* (severely ventralized)). (**i**–**l**) Post-embryonic morphology of control (**I**,**j**) and *chdA* morphant goldfish (**k**,**l**). (**j**,**l**) Ventral views of caudal regions of alizarin red-stained juvenile individuals of control (**j**) and *chdA* morphant individuals (**l**). (**m**–**t**) Morphology of late- and post embryonic stage common carp individuals (**m**,**n**: *chdA* morphants; **o**,**p**: *chdA* and *-B* double morphants). Black arrows, black arrowheads, white arrowheads, asterisks, and brackets indicate bifurcated fin folds, ectopically-accumulated blood, enlarged blood islands, expanded yolk extension, and malformation of fin folds, respectively. Scale bars = 0.5 mm (**a**–**h**), 1 cm (**i**–**l**), 1 mm (**m**,**o**,**q**–**t**).

To ensure appropriate comparison of the phenotypes of different teleosts under *chordin* gene-deficient conditions^20^, we first reproduced single and double *chordin* gene deficient goldfish for comparison with twin-tail goldfish and *dino* zebrafish at late- and post-embryonic stages (Fig. 2a–l and Supplementary Fig. S1). Wildtype goldfish *chdA* morphant embryos exhibit typical *chordin* mutant phenotypes (weakly ventralized or bifurcated fin fold) (Fig. 2a–f); in fact, a few morphants exhibited the same morphology as that of twin-tail goldfish at late embryonic stage (Supplementary Fig. S4). Moreover, the bifurcated fin fold in *chdA* morphant goldfish differentiated into a bifurcated caudal fin and its rays at the post-embryonic stage, showing almost identical morphology with the bifurcated caudal fin of twin-tail goldfish adult (Figs 1b and 2i–l). Furthermore, *chdB* morphant twin-tail goldfish exhibit severely ventralized embryonic features (Fig. 2g,h), akin to that of previously reported *dino* zebrafish mutants^12^,^14^. We also co-injected *chdA* MO reagent and *chdB* mRNA into wildtype goldfish (Supplementary Fig. S1a,b). The *chdA* morphant phenotype was rescued by *chdB* mRNA in a dose-dependent manner, consistent with previous experiments in which the micro-injection of *chdB* mRNA rescued the twin-tail goldfish phenotype^5^. Although the phenotype proportions varied between different embryo clutches, the same phenotypes were repeatedly reproduced in independent experiments involving the injection of 5 to 10 ng of *chdA* MO reagent into embryos (Supplementary Figs S1 and S5).

These late embryonic phenotypes are also consistent with embryonic gene expression patterns (Supplementary Fig. S6). The expression patterns of three marker genes were more ventralized in both the *chdA* mutant and morphant goldfish embryos than those in the respective controls (Supplementary Fig. S6a–c,e–g,i–k). In addition, *chdA* and *-B* double deficient goldfish embryos exhibit dramatic alterations in their gene expression patterns; the *gata2a* and *szl* gene expression areas encompassed an area wider than that of the ventral half of embryos, while the *foxb1a* positive region was restricted to a narrow region of the dorsal side (Supplementary Fig. S6d,h,l); this observation strongly suggests that the severe late embryonic phenotypes arise from the highly ventralized embryonic features (Fig. 2g,h). The similarity of the gene expression patterns and late embryonic phenotypes between *chdA* and *-B* double-deficient goldfish and previously reported *dino* zebrafish mutants indicate that our designed MO reagent clearly depleted *chordin* genes transcripts in goldfish (Fig. 2g,h and Supplementary Fig. S6d,h,l)^12^.

### Phenotype analysis of the *chordin* gene-deficient condition in common carp

**Figure 3:**
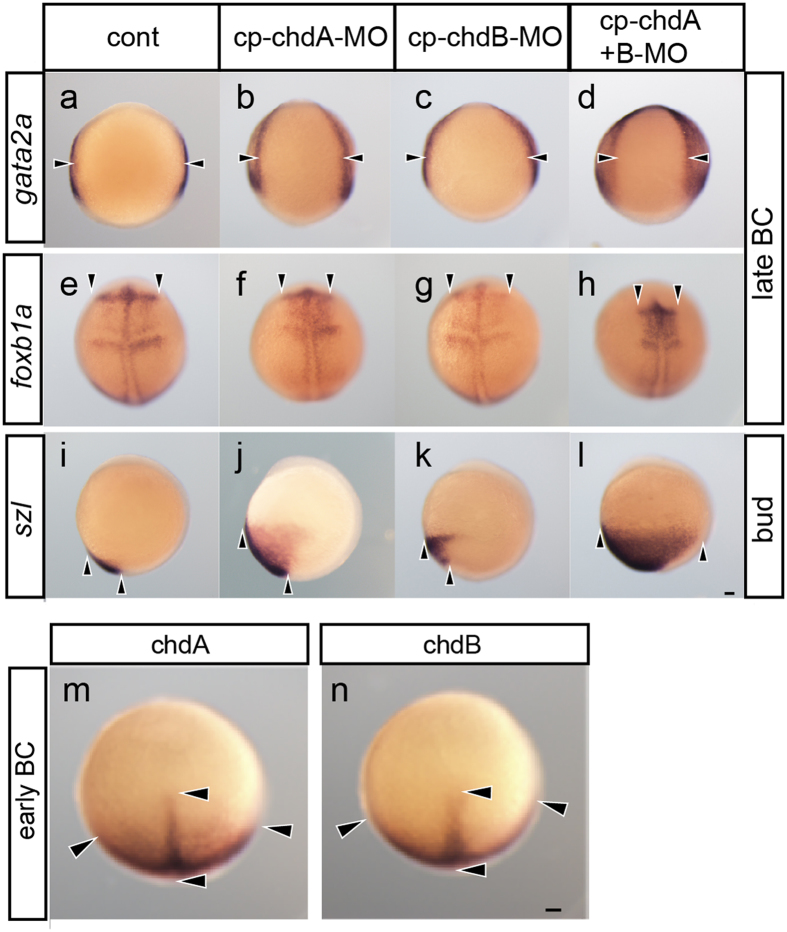
Comparison of gene expression patterns in *chordin* gene deficient common carp. Expression patterns of *gata2a* (dorsal view; **a**–**d**), *foxb1a* (**e**–**h**; dorsal view), and *szl* (**i**–**l**; lateral view) genes in control (**a**,**e**,**i**), *chdA*- (**b**,**f**,**j**), *chdB*- (**c**,**g**,**k**), and *chdA* and *-B* double morphant embryos (**d**,**h**,**l**). (**m**,**n**) Expression patterns of *chdA* and *-B* genes in the animal pole view of common carp embryos at the early blastopore closure stage (black arrowheads indicate areas of gene expression). Panels (**a**–**l**,**u**,**v**) are shown at the same magnification. Black arrowheads indicate areas of gene expression. Scale bars = 0.1 mm.

As may be expected from the phylogenetic proximity of goldfish and common carp (Fig. 1a), the phenotypes of *chordin* gene morphants of these species are similar. The *chdA* and *-B* single or double morphant common carp exhibit a range of ventralized phenotypes at the late embryonic stage (Fig. 2m–p, Supplementary Figs S7 and S8). In fact, the *chdA* and *-B* double morphant common carp phenocopied *dino* zebrafish and *chdA* and *-B* double deficient goldfish at the late embryonic stage; these embryos exhibit severely ventralized phenotypes with bifurcated fin folds (Fig. 3o,p, Supplementary Figs S7g,h and S8). Furthermore, these ventralized phenotypes of the *chdA* and *-B* single or double morphant consistent with their early embryonic expression patterns of the *gata2a*, *foxb1a*, and *szl* genes (Fig. 3a–l).

However, goldfish and common carp *chdA* morphant late embryos differ in the position of the bifurcated fin fold (Fig. 2e,f,m,n, Supplementary Figs S4 and S7). Unlike the *chdA* morphant goldfish, in which the bifurcated fin fold is located at a wide range of caudal levels (Fig. 2f and Supplementary Fig. S4), the bifurcated fin fold of *chdA* morphant common carp is restricted to the level around the yolk extension (Fig. 2m,n and Supplementary Fig. S7f). Moreover the bifurcated fin fold of the *chdA* morphant common carp does not differentiate into a bifurcated caudal fin at the post-embryonic stage (Fig. 2q–t); the bifurcated fin fold is unable to differentiate due to its overly anterior location (Fig. 2m,n and Supplementary Fig. S3d–i)^21^. Although we repeatedly examined the effects of various MO reagents and doses using three batches of embryos (the numbers of phenotyped embryos are provided in Supplementary Fig. S8), we did not observe common carp embryos with phenotypes resembling those of twin-tail and *chdA* morphant goldfish (i.e., a bifurcated fin fold at the caudal level without severe ventralization) (Fig. 2e,f and Supplementary Fig. S4). The results obtained using *chdA* morphant common carp suggest that the ventralized gene expression patterns do not reflect the late- and post-embryonic morphology of this species (Figs 2m,n and 3a–l), contrary to the findings in goldfish (Supplementary Fig. S6); these findings imply that the *chordin* gene-related developmental mechanisms differ between these teleosts, thereby affecting the penetrance of twin-tail morphology^9^,^15^,^16^.

### Overlapping expression patterns of *chdA* and *-B* genes in common carp

**Figure 4:**
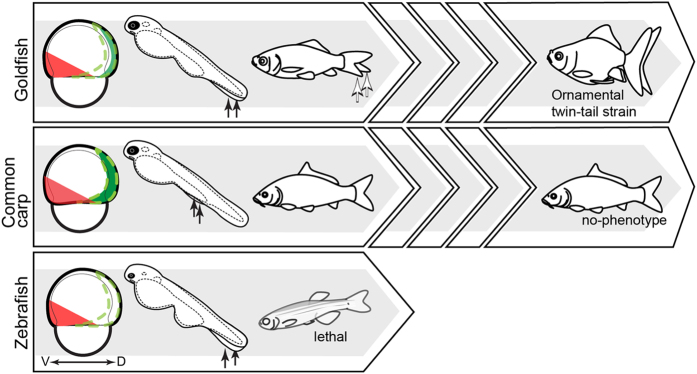
Schematic drawing of the hypothetical evolutionary consequences of *chordin* mutation. The three large arrow-shaped boxes on the left represent a single generation of goldfish (top), common carp (middle), and zebrafish (bottom). Drawings of early-, late, and post-embryonic phenotypes are shown for each species. The narrow arrow-shaped boxes shown for goldfish and common carp represent multiple generations. Black and white arrows within the arrow-shaped boxes indicate bifurcated fin folds and bifurcated caudal fins, respectively. Expression patterns of ventral markers and *chdB* genes are represented by red and dark green areas, respectively. The boundaries of the original expression patterns of goldfish *chdA* and the zebrafish *chordin* gene are indicated by green dashed lines. V: ventral; D: dorsal.

To further examine this possibility, we compared the *chdA* and *-B* gene expression patterns of common carp. Unlike goldfish, in which the *chdA* and *-B* exhibit spatially diverged expression patterns^9^, these genes in common carp showed completely overlapping expression patterns at the early blastopore closure stages (Fig. 3m,n); in fact, the use of probes designed against either the 3′- or 5′ UTR region revealed almost identical, overlapping expression patterns for these genes in early gastrula stage embryos (Supplementary Fig. S9). These data indicate that the *chdA* and *-B* genes are not sub-functionalized in terms of their expression patterns at the gastrula stage in common carp, although they are highly sub-functionalized in goldfish (Fig. 4)^9^. This further suggests that the *chdB* gene can compensate almost entirely for the loss of *chdA* in common carp at the early gastrula stage (Figs 3m,n, and 4). On the basis that the gastrula and its subsequent embryonic stages are of developmental importance for the formation of the caudal embryonic region^22^, we may anticipate that differences in *chordin* gene expression patterns between goldfish and common carp may be responsible for the substantial spatial and morphological differences in caudal fin phenotypes observed between these two species (Figs 2k,l,s,t and 3 and Supplementary Fig. S6). In other words, while the effects of *chdA* deficiency readily manifest as twin-tail morphology in goldfish (Figs 2e,f,k,l and 4, and Supplementary Fig. S6b,f,j)^9^, the effects may be compensated and buffered in common carp due to the overlapping expression pattern of *chdB* (Figs 2q–t,3m,n and 4 and Supplementary Fig. S9)^23^.

## Discussion

The MO knockdown data presented here suggest that the recently duplicated *chordin* paralogous genes may have facilitated genetic fixation of twin-tail morphology in the goldfish lineage, consistent with our previous findings^9^. In contrast, the presence of paralogous genes may have impeded appearance of this morphology in the common carp lineage (Figs 1a and 4). It is expected that cis- and/or trans-regulatory factors and divergence of coding sequences may contribute to the differences in overlapping expression patterns of *chordin* paralogues between goldfish and common carp. To identify the major factor underlying the differentiated overlapping expression patterns of these teleosts, we will need to make further detailed comparisons of whole genome information and detailed observation of their molecular developmental processes, a goal complicated by the intricate nature of the *chordin* gene-related gene regulatory networks^15^,^16^,^24^.

Based on the knockdown data, we can predict that, even if the common carp *chdA* gene possessed a stop codon mutation equivalent to goldfish *chdA*^*E127X/E127X*^ (Supplementary Fig. S1), the stop codon mutation would not be detected and fixed in the population by breeders because its effect would be masked by the overlapping expression pattern of *chdB* (Figs 1a and 4)^23^,^25^. Moreover, this hypothetical mutated common carp *chdA* gene would most likely become a pseudogene, in accordance with the general fate of duplicated genes present in the zebrafish and medaka genomes^26^,^27^; such pseudogenization would result in common carp becoming equivalent to zebrafish and medaka in terms of the number of *chordin* genes.

Taken together, it can be concluded that goldfish underwent a series of evolutionary events (duplication of the *chordin* gene, sub-functionalization of *chordin* gene expression patterns, and selection of morphological features that enabled the fixation of the *chdA*^*E127X*^ allele in the goldfish population) in an order which enabled drastic morphological change; although common carp underwent *chordin* gene duplication and was subjected to the same type of selective pressure as goldfish, the absence of a sub-functionalization event prevented the emergence of twin-tail morphology (Figs 1 and 4).

Finally, comparison of the results presented here with phenotypic and genome data from other vertebrate species promises to provide insights into how large-scale morphological changes can occur in a certain lineage. Our previous anatomical analyses indicated that the bifurcated caudal fin contains bifurcated axial skeletal structures, which are located on the sagittal plane of the body in all other vertebrates^9^. Other examples of novel bilaterally-positioned internal skeletal morphological characteristics with homologous structures located on the sagittal plane of the body include bilaterally-located nasal capsules and paired fins in jawed vertebrates; these morphological characteristics are known to have emerged after the divergence between jawed and jawless vertebrates and be present on the sagittal plane in jawless vertebrates^28^,^29^,^30^,^31^,^32^. However, such evolutionary re-organizations of internal skeletal architecture in the vertebrate lineage may not occur over short time periods, even in ornamental domesticated animals. For example, although genome sequence analyses of domesticated dog and pigeon strains revealed that mutations of several genes altered various phenotypic features, including ornamental morphology and body size, the basic body architecture of these organisms remained conserved^3^,^4^. As such, the genome of these species may not be amenable to large scale re-organization of skeletal architecture, even if breeders desired highly diverged ornamental morphology (for example, “twin-tailed dogs” or “twin-headed pigeons”). Gene duplications may provide an opportunity for genetic mutations that alter the basic body architecture of vertebrates to occur, as it is generally accepted that gene duplication increases phenotypic complexity during long-term evolution^25^,^26^,^33^,^34^.

However, here we have shown that while gene duplication played a positive role in goldfish morphological diversification, it played a negative role in that of common carp. Moreover, although certain trout and Xenopus species underwent lineage-specific genome duplications, the absence of appropriate selective pressure on these species presumably prevented drastic morphological diversification^35^,^36^. Our results imply that the sequential occurrence of evolutionary events (duplication, sub-functionalization, and subsequent strong selective pressure) in a certain order, as opposed to the accumulation of random, isolated events, might enhanced drastic large-scale morphological changes involving re-organization of skeletal architecture in the lineage of the goldfish. In other words, the conserved basic body architecture of vertebrate species and rarity of drastic morphological changes may be explained by the low probability of evolutionary events occurring in the required order.

## Methods

### Goldfish strains and common carp

Wildtype and twin-tail goldfish were purchased from an aquarium fish agency and breeder in Taiwan. To avoid confusion derived from complicated goldfish nomenclature systems^7^,^8^, goldfish individuals with a slender body and a single caudal fin and ornamental goldfish with a bifurcated caudal fin (as described in ref. 8) were collectively designated as wildtype- and twin-tail goldfish, respectively. Wildtype goldfish individuals were genotyped at the *chdA* locus. Goldfish individuals with *chdA*^*wt/wt*^ and *chdA*^*wt/E127X*^ alleles were separately maintained in our aquarium facilities. The genotyping method was based on that of a previous report^9^. Common carp were obtained from local breeders or were directly caught from the ErRong river water system in Yilan prefecture, Taiwan.

### Fish embryos and juveniles

The artificial fertilization procedures for goldfish and common carp are very similar. Sperm was extracted from male individuals and preserved in Modified Kurokura’s extender 2 solution at 4 °C^37^. Eggs were squeezed from mature female individuals onto Teflon-coated dishes. Artificial fertilization was performed using dry methods. Fertilized eggs were placed in 9cm Petri dishes containing tap water (23–24 °C). Petri dishes containing approximately 50 individuals were incubated at 24 °C until observation and harvesting. Embryonic and juvenile stages were determined using the goldfish staging table before harvesting^21^,^38^. All experiments were carried out in accordance with the approved guidelines. All experimental protocols were approved by Institutional Animal Care & Utilization Committee, Academia Sinica.

### Observation of the development of the goldfish caudal fin fold

Backcross progenies derived from the cross of a twin-tail goldfish (*chdA*^*E127X/AE127X*^) and a wildtype goldfish individual of the *chdA*^*wt/E127X*^ genotype were phenotyped and separately maintained in a 9 cm dish. More than 20 individuals were continuously maintained, of which 18 individuals were photographed at 2, 4, and 18 days post fertilization (dpf) under stereomicroscopy (SZX16; Olympus).

### Molecular cloning and sequencing

Homologues of *gata2a*, *fox1ba*, *szl*, *chdA*, and *-B* were isolated from embryonic cDNA. Total RNA was extracted from gastrula and segmentation stage embryos using TRIzol Reagent (Ambion). Degenerate and specific PCR primers were designed on the basis of conserved amino-acid sequences, goldfish RNA-seq^9^, and common carp genome sequence data^17^. Primers used to clone goldfish genes are listed in Table S1. PCR fragments amplified using these primers were isolated and purified, and then ligated into vectors using the TOPO TA Cloning Kit Dual Promoter (Invitrogen), T&A Cloning Vector Kit (Yeastern Biotech) or PGEM-T Easy Vector system (Promega). The resulting vectors were used to transform *Escherichia coli* DH5α. At least 12 clones were selected from each population for sequencing. The sequenced cDNA fragments were used as backbones to obtain almost complete sequences by PCR, with specific primers and the GeneRacer kit (Invitrogen), according to the manufacturer’s protocol (Supplementary Table S1). The isolated genes were identified by generating multiple amino acid alignments of goldfish, common carp, and orthologous genes of zebrafish and cave fish. The phylogenetic relationship between *chdA* and *-B* were investigated by reconstructing a maximum likelihood tree using MEGA5 (Supplementary Fig. S2). Sequences isolated from goldfish and common carp in this study have been deposited in the NCBI under accession numbers LC092194-LC092200.

### Morpholino injection

Antisense morpholino oligonucleotides (MO) (Gene Tools) were resuspended in water as a 1 mM stock solution and diluted in 0.2 M KCl solution before use to the appropriate concentration. Phenol Red (Sigma) was added as an indicator at a final concentration of 0.05%. A microinjector (Eppendorf Femtojet; Eppendorf) was used to inject 2–4 nl of MO solutions into the yolk of fertilized eggs maintained on Petri dishes at the 1–2 cell stage. The injected embryos were incubated at 24 °C. The sequence of the MOs used for blocking translation are as follows: *ca-chdA-MO*: GCGCTGACAGAGACGACGAAACCAA; *ca-chdB-MO*: ACAGCACTCGCGCAGCTTCCATTCC; *cy-chdA-MO*: GAACGCTTCCTCTGCGCCAAAACGC; *ca-chdB-MO*: AACTTCTGATCTAACTCTCCTGCGC (Supplementary Fig. S1).

### Phenotype observations of morpholino-injected specimens

The morpholino-injected specimens were phenotyped at the late embryonic stage. Embryos at 2–3 dpf were categorized based on morphology at the posterior half body into four types: wild-type; weakly-ventralized; bifurcated fin fold; severely-ventralized. The embryonic phenotypes were defined on the basis of earlier descriptions of zebrafish *dino* mutants and goldfish backcross segregants^9^,^12^,^14^. The post-embryonic stage specimens were observed at both pelvic fin ray- and juvenile stages^21^. For ease of observation of exoskeletal tissues (especially fin rays), the post-embryonic stage specimens were stained by alizarin red based on a method described in a previous report^21^. All morpholino-injected specimens were observed under stereomicroscopy (SZX16 and SZ16; Olympus).

### *In situ* hybridization

Digoxigenin-labelled anti-sense RNA probes were produced using PCR products as template, and the SP6/T7 RNA polymerase Riboprobe Combination System (Promega), in accordance with the manufacturer’s instructions. The probes were subsequently purified using mini Quick Spin RNA Columns (Roche). Primer sets used for the amplification of the PCR fragments are listed in Table S1. PCR products corresponding to a region including the relevant 5′ or 3′ untranslated regions (UTRs) were used to generate probes for analyses of *gata2a*, *fox1ba*, and *szl* expression patterns. To avoid non-specific hybridization resulting from the high similarity of the *chdA* and *chdB* transcripts, anti-sense probes against these *chordin* genes were synthesized from sequences encompassing the 3′ and 5′ UTRs.

Whole-mount *in situ* hybridization was performed as previously described^39^, with minor modifications. Fish embryos were fixed with 4% paraformaldehyde in PBS overnight. Gastrula stage embryos were fixed and then dechorionated using forceps. Segmentation and pharyngeal stage embryos were dechorionated before fixation through pronase treatment. After fixation and dechorionation, embryos were dehydrated with methanol. Dehydrated embryos were re-hydrated with PBT and re-fixed with 4% paraformaldehyde in PBS. Embryos were subsequently treated with Proteinase K for 20 min, and then re-fixed. Pre-hybridization and hybridization were performed at 65 °C for a period between 1 h and overnight. The samples were washed sequentially with 66% formamide/2 × SSCT at 65 °C for 30 min, 33% formamide/2 × SSCT at 65 °C for 30 min, 2 × SSCT at 65 °C for 15 min, and 0.2 SSCT at 65 °C for 30 min (this last wash was repeated twice). The samples were then incubated in blocking solution (PBS, 10% heat-inactivated goat serum (Roche), 0.1% Tween-20) for 1 h, before being incubated with a 1/4,000–1/8,000 volume of anti-digoxigenin-AP Fab fragments (Roche) at room temperature for 4 h or at 4 °C overnight. Samples were washed four times with blocking solution at room temperature for 25 min each. Signals were detected using BCIP/NBT Color Development Substrate (Promega). The reaction was stopped by washing the samples with PBS. To ensure accurate comparison of gene expression levels, the compared embryos were treated under identical conditions.

## Additional Information

**Accession codes:** Sequences isolated from goldfish and common carp in this study have been deposited in the NCBI under accession numbers LC092194-LC092200. 

**How to cite this article**: Abe, G. *et al.* Open and closed evolutionary paths for drastic morphological changes, involving serial gene duplication, sub-functionalization, and selection. *Sci. Rep.* **6**, 26838; doi: 10.1038/srep26838 (2016).

## Supplementary Material

Supplementary Information
