# Supplementary material for: Open and closed evolutionary paths for drastic morphological changes, involving serial gene duplication, sub-functionalization, and selection
Source: Sci Rep. 2016 May 25;6:26838. doi: 10.1038/srep26838 (PMC4879570; doi:10.1038/srep26838)
Supplement: Supplementary Information [file srep26838-s1.pdf]

Title: Open and closed evolutionary paths for drastic morphological changes, involving serial gene duplication, sub-functionalization, and selection

Gembu Abe[1, 2], Shu-Hua Lee[1], Ing-Jia Li[1], Chun-Ju Chang[1], Koji Tamura[2] and Kinya G. Ota[1*]

[1]Laboratory of Aquatic Zoology, Marine Research Station, Institute of Cellular and Organismic Biology, Academia Sinica, Yilan, 26242, TAIWAN.

[2]Laboratory of Organ Morphogenesis, Department of Developmental Biology and Neurosciences, Graduate School of Life Sciences, Tohoku University, Aobayama Aoba-ku, Sendai 980-8578, JAPAN.

*otakinya@gate.sinica.edu.tw

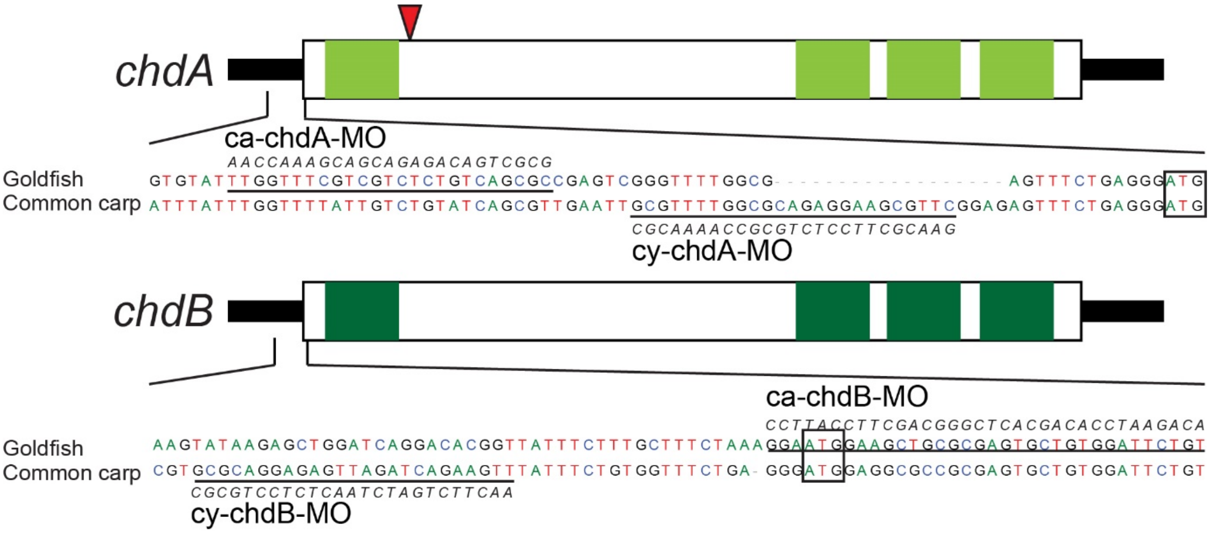

Supplementary Figure S1: Schematic views of ChdA and -B proteins and partial sequences of *chdA* and –*B* mRNA with morpholino-binding regions. The positions of cysteine-rich domains are indicated by light green (*chdA*) and dark green (*chdB*) boxes. The red arrowhead indicates the position of the stop codon mutation (*chdA*^{E127X} allele) reported in twin-tail goldfish (Abe et al., 2014). Four morpholino oligonucleotides (ca-chdA-MO, ca-chdB-MO, cy-chdA-MO, and cy-chdB-MO) were designed for blocking the translation of goldfish (ca) or common carp (cy) *chdA* and -*B* genes.

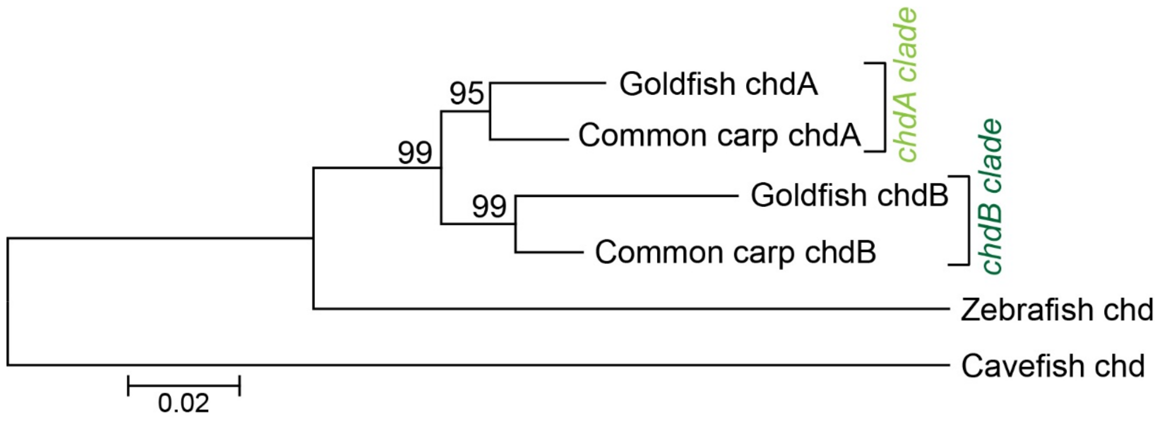

Supplementary Figure S2: Phylogenetic relationship of *chordin* genes. A phylogenetic tree of the *chordin* genes from goldfish, common carp, zebrafish, and cavefish was reconstructed by the maximum likelihood method based on a multiple alignment of amino acid sequences, using the MEGA5 program. The phylogenetic relationship between *chdA* and –*B* clades was clearly separated with high support values (over 95%). Chordin sequence accession numbers from DDBJ/EMBL/NCBI and transcript IDs from Ensemble Genome Browser are as follows: Zebrafish chordin, NM_130973; Cavefish chordin, ENSAMXT00000012078; Goldfish chdA, BAO51895.1; Goldfish chdB, BAO51897; Common carp chdA, LC092194; Common carp chdB LC092195. The final dataset contained a total of 957 positions.

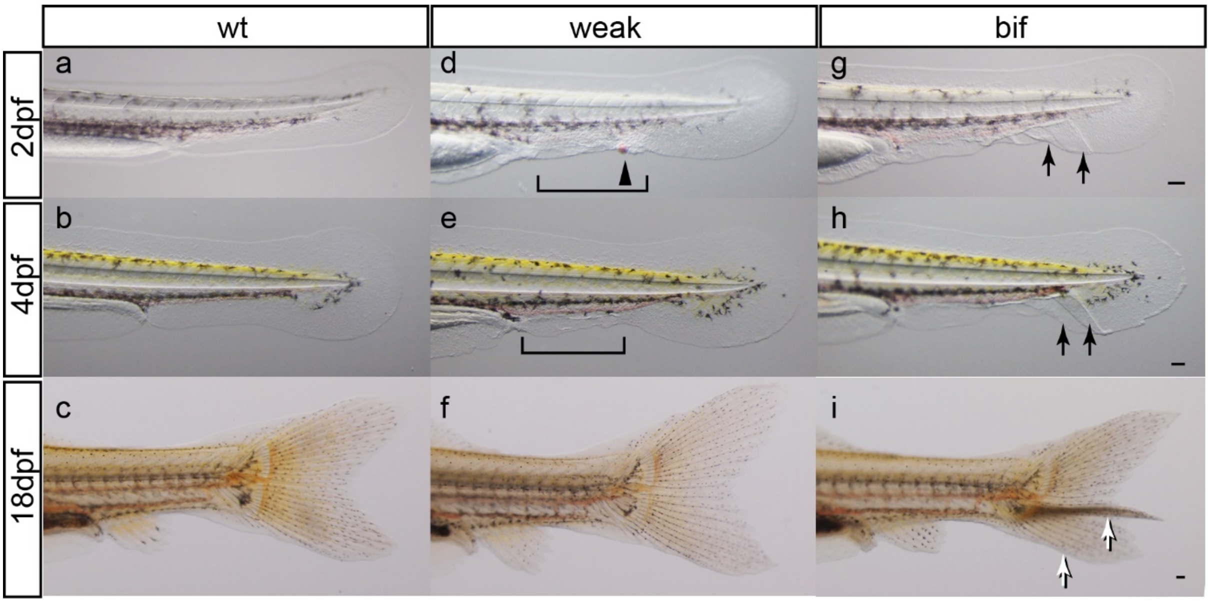

Supplementary Figure S3: Post-hatching development of the goldfish caudal region. (**a-c**) Wildtype (single-tail) goldfish individual. (**d-f**) Weakly ventralized goldfish individual bearing the *chdA*$^{E127X/E127X}$ locus. (**g-i**) Bifurcated caudal fin individual bearing the *chdA*$^{E127X/E127X}$ locus. The development of caudal regions was traced in identical individuals at 2, 4, and 18 days post-fertilization (dpf). Black arrows, white arrows, black arrowheads, and brackets indicate bifurcated fin folds, bifurcated caudal fins, ectopic accumulation of blood cells, and fin fold areas showing developmental malformations, respectively. In the weakly ventralized individual, the malformed area and ectopic accumulation of blood cells ultimately reduced in size and disappeared, while the bifurcated fin fold finally differentiated into a bifurcated caudal fin, at 18 dpf. Scale bar = 0.1mm. Upper (**a**, **d**, **g**), middle (**b**, **e**, **h**), and lower (**c**, **f**, **i**) panels are all presented at the same magnification.

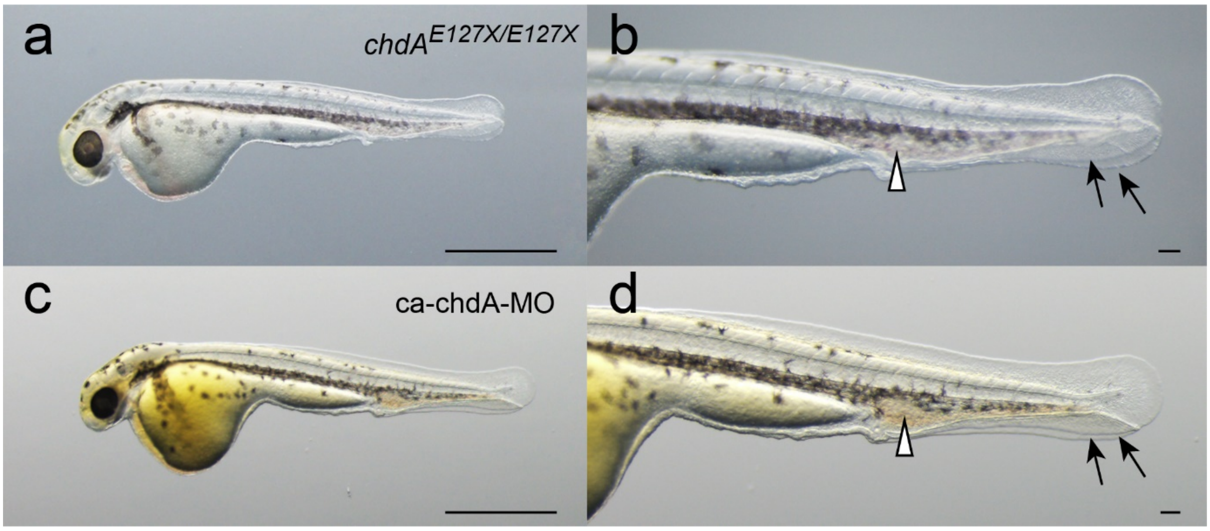

Supplementary Figure S4: Comparison of chdA mutant and morphant goldfish embryos.

(**a**, **b**) Late pharyngular stage embryo of twin-tail goldfish (*chdA* mutant; *chdA*$^{E127X/E127X}$). (**c**, **d**) Late pharyngular stage embryo of *chdA* morphant goldfish. Panels **b** and **d** are magnified views of panels **a** and **c**, respectively. White arrowheads and black arrows indicate enlarged blood islands and bifurcated fin folds, respectively. Scale bars = 1mm (**a**, **c**), 0.1mm (**b**, **d**).

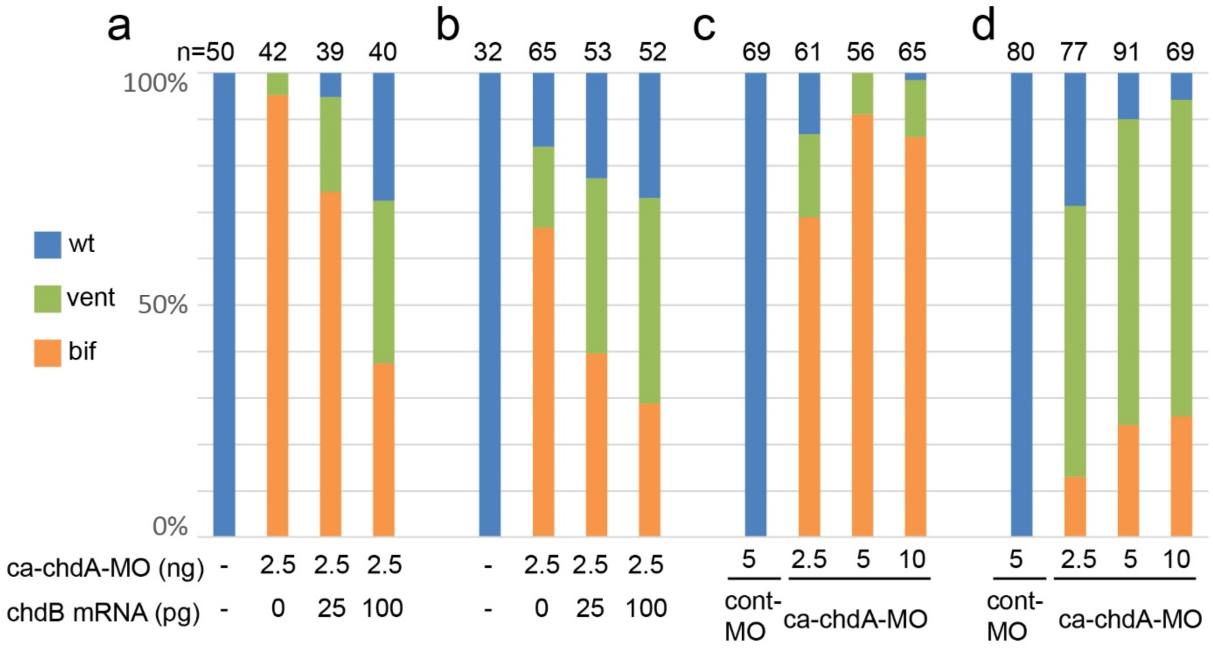

Supplementary Figure S5: Phenotype analyses following the co-injection of *chdA* MO and *chdB* mRNA into wildtype goldfish embryos. Co-injection of *chdA* MO and *chdB* mRNA (**a** and **b**). The *chdA* morpholino knock down phenotype was rescued by *chdB* mRNA in a dose-dependent manner (**a** and **b**), suggesting that *chdA* MO depleted *chdA* function in goldfish during early embryogenesis. Dose-dependent effect of *chdA* MO injection (**c** and **d**). The effects of injecting 5 ng or 10 ng *chdA* MO per embryo were similar and reproducible in both clutches (**c** and **d**), suggesting that MO concentrations of 5 ng per embryo are sufficient to induce the twin-tail phenotype. Embryos of each experiment (a-d) were derived from different sets of parents. Phenotypes of embryos were categorized as follows: i) wildtype (wt); ii) weakly-ventralized (weak), and iii) bifurcated fin fold (bif). These criteria were based on previous phenotypic descriptions[9].

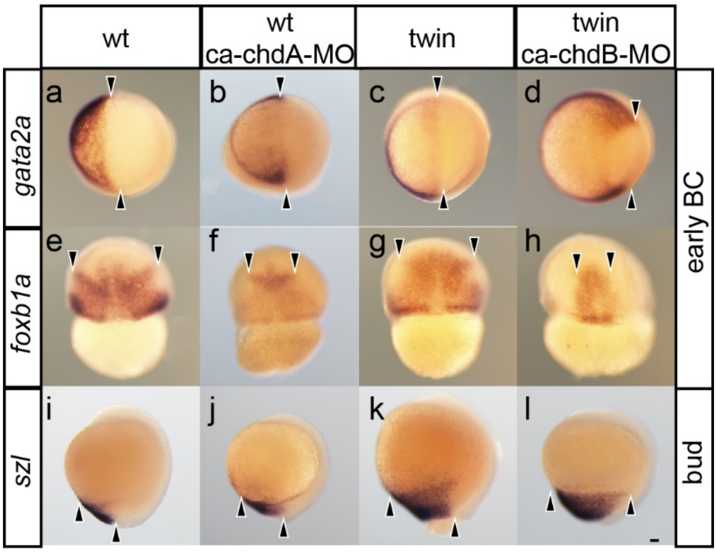

Supplementary Figure S6: Comparison of gene expression patterns of *chordin* gene deficient goldfish. Expression patterns of *gata2a* (animal pole view, **a**-**d**), *foxb1a* (dorsal view, **e**-**h**), and *szl* (lateral view, **i**-**l**). Expression patterns of *gata2a*, *szl*, and *fox1ba* in wildtype goldfish embryos (**a**, **e**, **i**), *chdA* morphant goldfish (**b**, **f**, **j**), twin-tail goldfish (**c**, **g**, **k**), and *chdB* morpholino-injected twin-tail goldfish embryos (**d**, **h**, **l**) are shown. Areas of gene expression are indicated by black arrowheads. All panels are shown at the same magnification. Scale bars = 0.5mm.

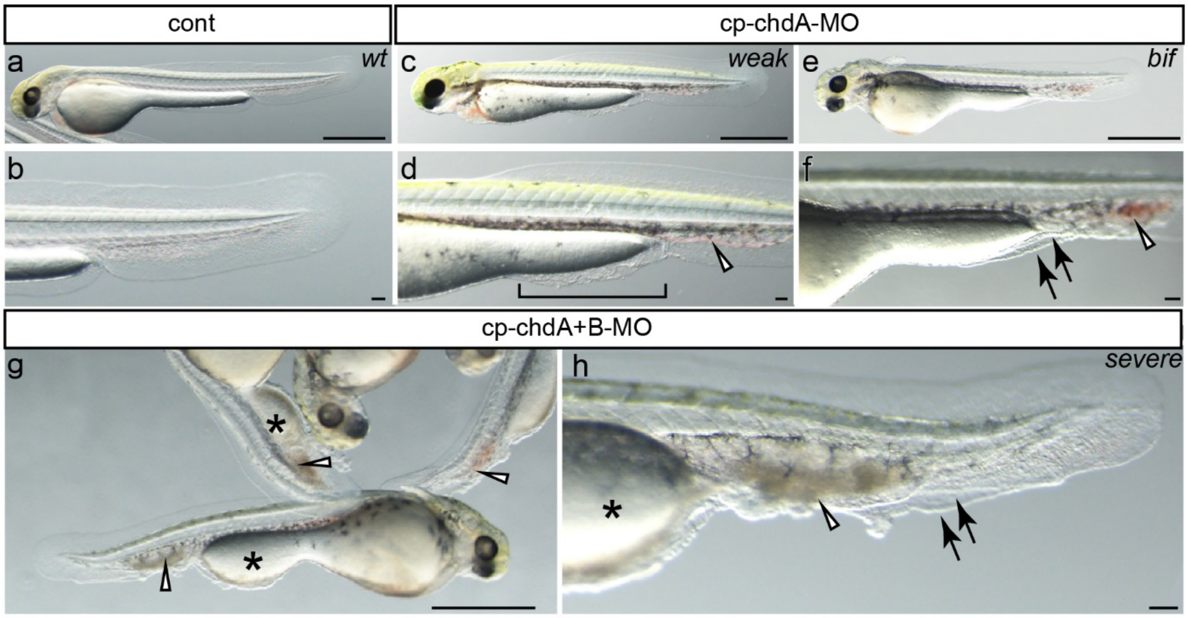

Supplementary Figure S7: Common carp morphant phenotypes. (**a-h**) Morphology of late stage embryos of control (**a**, **b**), *chdA* morphant (**c-f**), and *chdA* and –*B* double morphants (**g**, **h**). Panels **b**, **d**, **f**, and **h** are magnified views of panels **a**, **c**, **e**, and **g** respectively. Black arrows, white arrowheads, asterisks, and brackets in panels **d**, **f**, **g**, and **h** indicate bifurcated fin folds, enlarged blood islands, expanded yolk extensions, and malformed fin fold, respectively. Panels **e**, **f**, **g**, and **h** are identical to panels **a**, **b**, **c**, and **d** in **Fig. 4**, respectively. Scale bars=1mm (**a, c, e, g)**, 0.1mm (**b, d, f, h**).

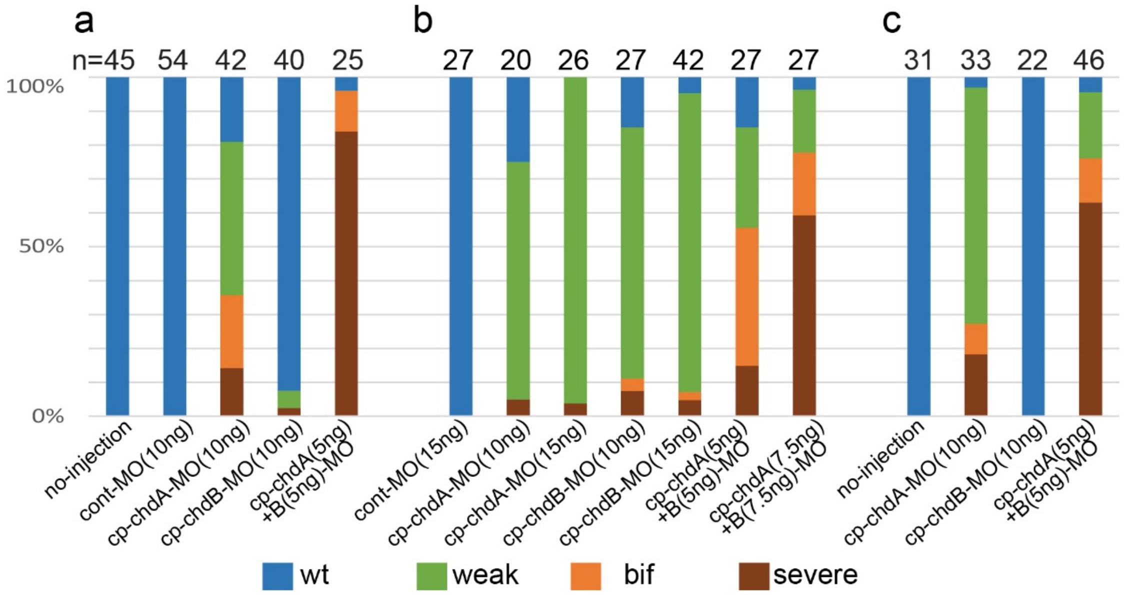

Supplementary Figure S8: Proportions of observed phenotypes in *chordin* gene morphant common carp late embryos. Data from three different batches are shown (**a**-**c**). Higher doses of MO reagent(s) (15 ng/embryo) were injected in the second batch of experiments (**b**). Injected morpholino reagent(s) and dose are indicated at the bottom of the graphs. The total number of phenotyped *chdA*, -*B*, and *A*+*B* double morphants in the first to third experiments (**a**-**c**) were 121, 131, and 125, respectively.

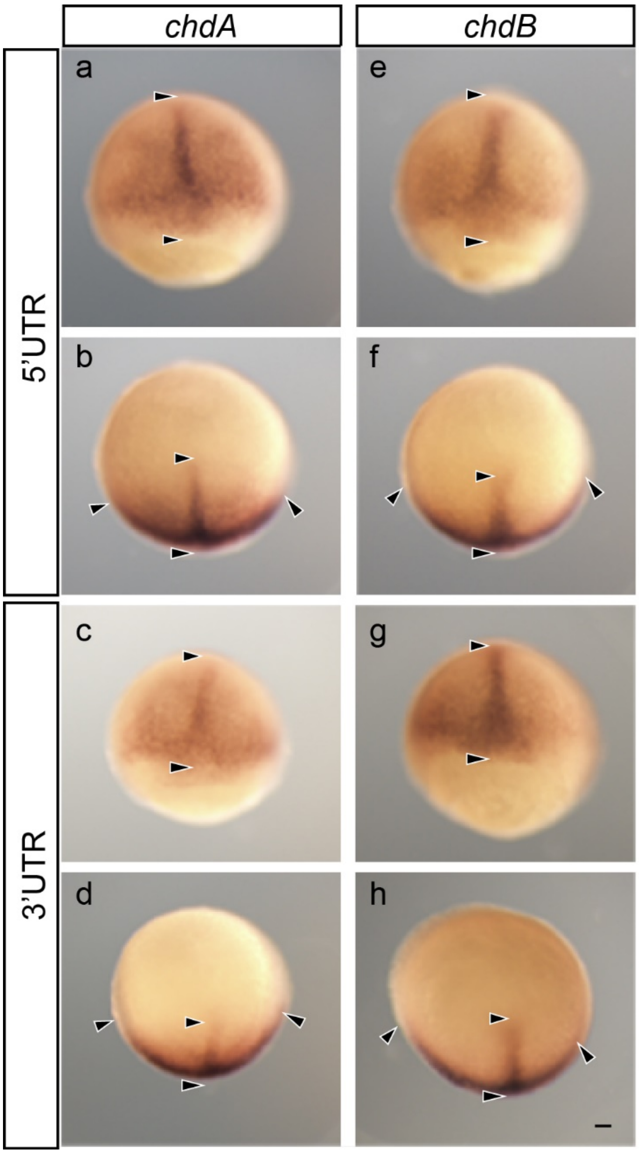

Supplementary Figure S9: Comparison of *chdA* and –*B* gene expression patterns in common carp. Expression patterns of *chdA* (**a**-**d**) and –*B* (**e**-**h**) genes were examined from dorsal (**a**, **e**, **c**, **g**) and animal pole (**b**, **f**, **d**, **h**) views in early blastopore closure stage embryos, using probes designed against different cDNA sequence regions; 5'UTR (**a**, **b**, **e**, **f**) and 3'UTR (**c**, **d**, **g**, **h**). Black arrowheads indicate gene expression areas. Scale bar = 0.1mm. All panels are shown at the same magnification.

Supplementary Table S1: Primer list

species	gene	primer names	sequence (5' -> 3')
goldfish and common carp	*foxb1a*	foxb1a-f1	CCYAGACCCGGGAGAAACAC
goldfish and common carp	*foxb1a*	foxb1a-f2	AGAAACCGCCGTACTCCTACA
goldfish and common carp	*foxb1a*	foxb1a-r1	CTCTTGCCTTGATTGTATAGCCA
goldfish and common carp	*foxb1a*	foxb1a-r2	TGTCAGTGCACCGCGACC
goldfish and common carp	*gata2a*	sp6p-gata2a-f1	CAGTGAATTGATTTAGGTGACACTATAGAAGTGA TGGAGGTCGCGGCCGATCAGTCT
goldfish and common carp	*gata2a*	t7p-gata2a-r1	CAGTGAATTGTAATACGACTCACTATAGGGAGAG TGAGGCTCGGCCCAGGCCGGA
common carp	*chdA*	cc-chdA-5rr1	GTGCGACGCCGCTGGAGCTCGG
common carp	*chdA*	cc-chdA-5rr2	CGTGATGGAGAAGGTCAGGCTCGTTCGAGCAA
common carp	*chdA*	cc-chdA-gf2	AAACAGGACTGTCCCGATCCGAC
common carp	*chdB*	cc-chdB-5rr1	CCCGAGCGACGCCGCTGGAGTCCATT
common carp	*chdB*	cc-chdB-5rr3	ATCGGAGTCCAGGAAGGTGAGGA
common carp	*chdB*	cc-chdB-5rr4	GAGCGACGCCGCTGGAGTCCATTAC
common carp	*chdB*	cc-chdB-gf2	AAACAGGATTGTCCCGATCCGTC
common carp	*chdA and -B*	cc-chd-sf1	GCAACTCGGGGAGAAAGGAGG
common carp	*chdA and -B*	cc-chd-sr1	TGTTCTCCTCAGAGCTGATGTAGG
common carp	*chdA*	Carpio-chdA-SP6-F3	CAGTGAATTGATTTAGGTGACACTATAGAAGTGG GTGGGAGAAATGAAGTG
common carp	*chdA*	Carpio-chdA-T7R	TAATACGACTCACTATAGGGAGACAGACTGACAA CATTAGGTGC
common carp	*chdB*	Carp-chdB-SP6-F2	CAGTGAATTGATTTAGGTGACACTATAGAAGTGG CACCTGGCACAACATGGCATCC
common carp	*chdB*	Carp-chdB-T7-R1	TAATACGACTCACTATAGGGAGAGGATTTGAGTG TAAATATTAAGGATTGTAATGAC
common carp	*chdA*	Carp-chdA-SP6-5'race-insitu	CAGTGAATTGATTTAGGTGACACTATAGAAGTGA TCAGTGCGCACTGAC
common carp	*chdA*	Carp-chdA-T7-5' race-insitu	TAATACGACTCACTATAGGGAGAGATGTAGGATC TGTCATTG
common carp	*chdB*	Carp-chdB-SP6-5'race-insitu	CAGTGAATTGATTTAGGTGACACTATAGAAGTGC TGTATAAGACCCCGT
common carp	*chdB*	Carp-chdB-T7-5' race-insitu	TAATACGACTCACTATAGGGAGAGTGAATCTGGC CCGAGCG
common carp	*szl*	Carp-sizzled-SP6-F2	CAGTGAATTGATTTAGGTGACACTATAGAAGTGG TCAGGATCATGCATCTGTCT
common carp	*szl*	Carp-sizzled-T7-R880	TAATACGACTCACTATAGGGAGAGAGCCACGGG AACAGACTGG
common carp	*chdA*	Carp-chdA-full length-Fwd1	CACATTCACTGAGCTATATCCAGCAGAACCTCGC
common carp	*chdA*	Carp-chdA-full length-Rev1	TTTTAAAACTTTGTTTATTCATCATAATTAATAAAT ATGC
common carp	*chdB*	Carp-chdB-full length-Fwd2	CCCATCCAGCCAGAGACAGAACCCTTGATA
common carp	*chdB*	Carp-chdB-full length-Rev2	CATGTGATTGTTACAGGTGTGATGATGTATG
